# Risk Prediction of Cardiovascular Complications in Pregnant Women With Heart Disease

**DOI:** 10.5935/abc.20160028

**Published:** 2016-04

**Authors:** Luciana Carvalho Martins, Claudia Maria Vilas Freire, Carolina Andrade Bragança Capuruçu, Maria do Carmo Pereira Nunes, Cezar Alencar de Lima Rezende

**Affiliations:** 1 Universidade Federal de Minas Gerais - UFMG, Belo Horizonte, MG - Brazil; 2 Maternidade Odete Valadares - Fundação Hospitalar do Estado de Minas Gerais, Belo Horizonte, MG - Brazil

**Keywords:** Cardiovascular Diseases / complications, Pregnant Women, Risk Factors, Heart Failure, Arrhythmias, Cardiac, Rheumatic Heart Disease

## Abstract

**Background:**

Heart disease in pregnancy is the leading cause of non- obstetric maternal death. Few Brazilian studies have assessed the impact of heart disease during pregnancy.

**Objective:**

To determine the risk factors associated with cardiovascular and neonatal complications.

**Methods:**

We evaluated 132 pregnant women with heart disease at a High-Risk Pregnancy outpatient clinic, from January 2005 to July 2010. Variables that could influence the maternal-fetal outcome were selected: age, parity, smoking, etiology and severity of the disease, previous cardiac complications, cyanosis, New York Heart Association (NYHA) functional class > II, left ventricular dysfunction/obstruction, arrhythmia, drug treatment change, time of prenatal care beginning and number of prenatal visits. The maternal-fetal risk index, Cardiac Disease in Pregnancy (CARPREG), was retrospectively calculated at the beginning of prenatal care, and patients were stratified in its three risk categories.

**Results:**

Rheumatic heart disease was the most prevalent (62.12%). The most frequent complications were heart failure (11.36%) and arrhythmias (6.82%). Factors associated with cardiovascular complications on multivariate analysis were: drug treatment change (p = 0.009), previous cardiac complications (p = 0.013) and NYHA class III on the first prenatal visit (p = 0.041). The cardiovascular complication rates were 15.22% in CARPREG 0, 16.42% in CARPREG 1, and 42.11% in CARPREG > 1, differing from those estimated by the original index: 5%, 27% and 75%, respectively. This sample had 26.36% of prematurity.

**Conclusion:**

The cardiovascular complication risk factors in this population were drug treatment change, previous cardiac complications and NYHA class III at the beginning of prenatal care. The CARPREG index used in this sample composed mainly of patients with rheumatic heart disease overestimated the number of events in pregnant women classified as CARPREG 1 and > 1, and underestimated it in low-risk patients (CARPREG 0).

## Introduction

Maternal mortality is still very high in Brazil. According to the Brazilian Unified Health System data bank (DATASUS), in 2007 the maternal mortality in Brazil was 77 per 100,000 live births. Heart disease in pregnancy is the first cause of non-obstetric maternal death and the fourth cause of maternal death in general.^1^ Diagnosing heart disease before or at the beginning of pregnancy is fundamental to assess the maternal-fetal risk, and has an impact on the patients' approach and therapeutic strategy. The other causes of maternal death are inherent to the condition, and, unlike heart disease, are usually unpredictable.^2,3^

Several studies have investigated the risk factors for adverse outcomes and cardiac complications during pregnancy in women with heart diseases.^4-6^ However, only a few Brazilian studies have assessed them.^7^ The present study had the following objectives: to establish the prevalence and etiology of heart diseases in pregnant women cared for at our referral center; to identify the most frequent maternal complications and their repercussions on maternal and perinatal outcomes; and to assess the risk predictors of cardiac complications that may influence maternal-fetal outcomes.

## Methods

In addition, this study assessed the maternal Cardiac Disease in Pregnancy (CARPREG) risk score, developed by Siu et al.,^4^ aiming at classifying the risk of pregnant women with heart disease and at observing the predictors of cardiac and neonatal complications in that population with characteristics different from those of the population studied by the authors of that risk score.

This study included pregnant women with heart disease followed up from prenatal care up to delivery and puerperium by the team of the High-Risk Pregnancy Care Sector, from January 2005 to July 2010. A total of 153 women were cared for at that sector during that period. This study was approved by the Committee on Ethics in Research, and all patients provided written informed consent.

All pregnant women were examined by the same cardiologist and underwent tests to confirm the diagnosis and to classify and assess the severity of the heart disease, such as Doppler echocardiography, electrocardiography and 24-hour Holter monitoring.

To analyze the risk predictors of maternal cardiac complications from this historical cohort, this study included only patients with complete information. Those with the following characteristics were excluded: miscarriage (fetal loss before the 20th week); delivery at other institutions; twin pregnancies; and peripartum cardiomyopathy developed in the puerperium period. Thus, 132 of 153 pregnant women with heart disease followed up at the sector were included.

### Variables assessed

The possible risk predictors of maternal cardiovascular complications assessed were as follows: age; parity; number of visits to the high-risk prenatal care (HRPC); HRPC beginning on the third trimester; maternal smoking; previous cardiac complications and previous surgical or clinical heart treatments; need to begin or change cardiac medication during pregnancy for patients who changed, at the most, one functional class during follow-up, or dose adjustment to abide by a follow-up protocol; valve prosthesis; New York Heart Association (NYHA) functional class ≥ III at the beginning of HRPC; left ventricular (LV) systolic dysfunction; associated preeclampsia or systemic arterial hypertension (SAH); left heart obstruction (LHO); and calculated CARPREG risk score. The following disorders were grouped as LHO: mitral stenosis with valve area < 2.0 cm^2^; aortic stenosis with valve area < 1.5 cm^2^; and LV outflow tract gradient > 30 mm Hg.

The following variables relating to the ongoing pregnancy were assessed: gestational age at the beginning of prenatal care and number of consultations; cardiac complications during pregnancy; invasive procedures required during prenatal care; NYHA functional classification; comorbidities; delivery type; hospital length of stay; and obstetric complications. The neonatal variables assessed were gestational age at the time of delivery and birth weight.

The prediction index of risk for complications associated with pregnancy in women with heart disease (CARPREG risk score) was retrospectively calculated for each patient. The variables associated with cardiovascular complications according to the CARPREG risk score are defined in Chart 1. Pregnant women are classified as CARPREG 0, 1 or > 1 in the presence of none, one, or more than one defined risk factor.^4^ The patients in this study were distributed into three groups: CARPREG 0, CARPREG 1 and CARPREG > 1, and the percentage of complications occurring in each group was compared to that predicted according to the original score: 5%, 27% and 75%, respectively.

**Chart 1:** CARPREG (Cardiac Disease in Pregnancy) risk score. NYHA: New York Heart Association.

**Predictors of cardiovascular events**	**Points**
Prior cardiac event (heart failure, transient ischemic attack, infarction prior to pregnancy) or arrhythmias	1
NYHA functional class at baseline > II or cyanosis	1
Left heart obstruction (mitral valve area < 2.0 cm2; aortic valve area < 1.5 cm2; and LV outflow tract gradient > 30 mm Hg)	1
Reduced systolic ventricular function (ejection fraction < 40%)	1

### Definition of outcomes

The cardiac complications were described according to the definitions proposed by Siu et al.^4^ The following cardiac complications were considered: death due to heart disease; heart failure with acute pulmonary edema (documented on chest X-ray or bilateral pulmonary rales on posterior chest auscultation on physical examination); acute myocardial infarction; sustained symptomatic tachyarrhythmia or bradyarrhythmia requiring treatment; worsening of at least 2 NYHA functional classes as compared to baseline; and need for emergency invasive procedures during pregnancy.

### Statistical analysis

The Statistical Package for the Social Sciences (SPSS 17, Inc., Chicago, IL, USA) software was used for statistical analysis. The continuous variables were presented as mean ± standard deviation, and the categorical ones, as frequency and percentage.

The variables assessed were compared between the pregnant women with cardiovascular complications in pregnancy and those with favorable outcomes by use of the chi-square test (categorical variables) or non-paired Student *t* test (continuous variables with normal distribution).

Univariate analysis and multivariate logistic regression were performed to identify the variables associated with cardiovascular complications in pregnancy. The criterion used to select the variables to the multivariate model was clinical relevance or p < 0.20 on univariate analysis. A p value < 0.05 was considered statistically significant.

## Results

The maternal age ranged from 16 to 45 years (mean: 27.59 ± 7.17). Regarding the number of gestations, 50 patients (37.88%) were on their first gestation, 38 (28.79%) were on their second gestation, and 44 (33.33%) had at least three gestations [15 (11.36%) were on their fifth pregnancy or more].

Only 34 patients (25.75%) initiated their HRPC follow-up on the first gestational trimester. Most patients (79; 59.85%) initiated their HRPC follow-up on the second trimester, while 19 patients (14.40%), on the third trimester.

The major heart disease diagnoses in the study population were: rheumatic heart diseases, 82 patients (62.12%); congenital heart diseases, 18 (13.65%); arrhythmias, 15 (11.36%); and mitral valve prolapse, 6 (4.54%). Cardiomyopathies of different causes and other cardiac diseases added up to 11 patients (8.33%).

Of the 82 pregnant women with rheumatic heart disease, 19 (23.17%) had valve prosthesis, of whom, 14 (73.68%) had normal functioning prostheses and 5 had residual dysfunction or associated lesion in other valves. The mitral biological prosthesis was the most frequently found (13; 68.42%), followed by mitral mechanical prosthesis (3; 15.79%). Two patients had mitral-aortic mechanical prostheses, and only one had an aortic mechanical prosthesis.

Of the 18 patients with congenital heart disease, 9 (50%) had a shunt defect (ventricular septal defect; atrial septal defect; atrioventricular septal defect; and patent ductus arteriosus), 50% of which had been surgically repaired before pregnancy. Regarding the LHO, one pregnant woman had a bicuspid aortic valve, and another had coarctation of the aorta and bicuspid aortic valve. None of those lesions was surgically corrected before pregnancy. Diseases of the pulmonary valve (pulmonary valve stenosis or double lesion) added up to 3 patients (16.7%). Regarding cyanotic heart diseases, 3 patients were followed up, 2 of whom had uncorrected Ebstein's anomaly and 1 had corrected tetralogy of Fallot. One patient with severe tricuspid regurgitation was observed.

Fifteen patients (11.36%) had arrhythmic heart disease, 8 of whom (53.33%) had supraventricular tachyarrhythmias (paroxysmal supraventricular tachycardia, atrial flutter or fibrillation). Four patients (26.67%) had bradyarrhythmia (atrioventricular block and bundle branch blocks), and 3 had other arrhythmias.

Twenty patients (15.15%) smoked 5 to 40 cigarettes per day (mean of 8.63 ± 8.95), of whom 31.5% smoked more than 10 cigarettes per day. Regarding the associated comorbidities, 23 patients (17.42%) had one as follows: type I diabetes, 2 patients; chronic obstructive pulmonary disease/asthma, 11; thyroid diseases, 4; nephropathy, 1; epilepsy, 3; dermatomyositis, 1; and megaesophagus, 1.

Of 132 pregnancies, 57 (43.18%) had cardiovascular complications prior to the ongoing pregnancy. Cardiac decompensation followed by arrhythmias was the most frequent complication.

Forty-six patients (34.85%) had LHO, 44 of whom (95.65%) had rheumatic mitral stenosis, with a mean valve area of 1.60 cm^2^, which was considered severe in 11 (25%).

On the first prenatal visit, only 4 patients (3.3%) were classified as NYHA functional class III, 3 of which (75%) had moderate or severe mitral stenosis associated with moderate mitral regurgitation. One patient had dilated cardiomyopathy.

At baseline, 2 patients had LV ejection fraction lower than 40%, 18 (13.63%) had it between 40% and 60%, and the remaining had it normal (≥ 60%).

### Adverse outcomes

Cardiovascular complications occurred in 30 (22.72%) pregnant women. Cardiac decompensation, diagnosed as a two-level worsening in NYHA functional class or worsening in patients with functional class III at baseline, was the most frequent complication: 15 cases (11.36%). Cardiac arrhythmias occurred in 9 (6.82%) patients. Four patients (3.03%) required invasive procedures during the pregnancy as follows: one stent implantation in aortic coarctation and 3 percutaneous balloon mitral valvoplasties for severe mitral stenosis. One patient with severe mitral and aortic regurgitation and nephrotic syndrome died suddenly in the post-delivery period (Table 1).

**Table 1:** Distribution of the pregnant women according to the occurrence of cardiovascular complications

**Cardiovascular complications**	**n (%)**
Arrhythmias	8 (26.67)
Stroke	2 (6.67)
Cardiac decompensation	15 (50.00)
APE	0
IE	0
Sudden death*	1 (3.33)
Need for invasive procedure	4 (13.33)
BMV	3 (75)
Ao stent	1 (25)

* Severe mitral regurgitation. APE: acute pulmonary edema; IE: infectious endocarditis; BMV: balloon mitral valvoplasty; Ao: aorta.

**Figure 1:**
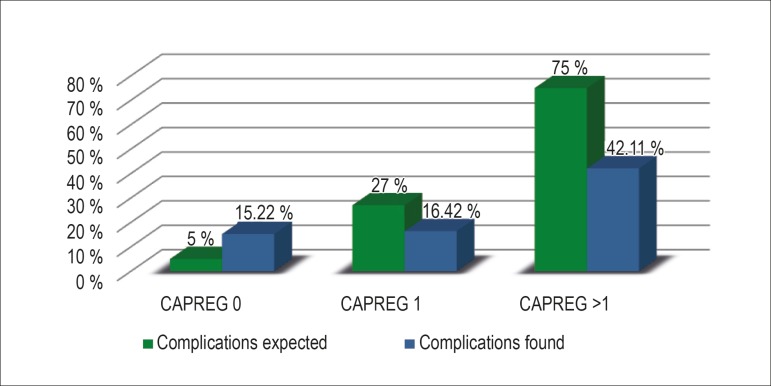
Percentage of complications expected during pregnancy according to the CARPREG risk score versus those found.

According to the CARPREG risk score, our population had the following percentages of complications: CARPREG 0, 46 patients (34.85%); CARPREG 1, 67 (57.76%); and CARPREG > 1, 19 (14.39%). The pregnant women classified as CARPREG > 1 had a significantly higher number of complications during pregnancy than those classified as the other CARPREG classes (p = 0.0013) (Table 2). The percentages of cardiovascular complications in the population studied, according to the CARPREG classes, were compared with those expected according to the original CARPREG risk score (Figure 1).

**Table 2:** Distribution of the gestations according to the occurrence of cardiovascular complications, as classified by the Cardiac Disease in Pregnancy (CARPREG) risk score

**Risk categories**	**Cardiovascular complications**		**p Value**
	**Present**	**Absent**	
CARPREG 0	15.2	_._8 8	0.013
CARPREG 1	16.4	83.6	
CARPREG>1	42.1	57.9	

Of the 132 pregnancies assessed to analyze risk predictors, the following were not cardiovascular complication predictors in pregnancy: maternal age (p = 0.071); number of visits to the HRPC (p = 0.344); maternal smoking (p = 0.327); SAH (p = 0.295); preeclampsia (p = 0.450); prenatal care beginning on the third trimester (p = 0.379); and valve prosthesis (p = 0.542). In addition, the non-cardiovascular diseases associated were not predictors of complication. On univariate analysis, the following factors were identified as risk predictors: need to initiate or change cardiac medication during pregnancy (p = 0.001); LHO (p = 0.018); cardiac complications prior to pregnancy (p = 0.002); ejection fraction <40% (p = 0.038); and NYHA functional class III on the first visit to the HRPC (p = 0.011) (Table 3).

**Table 3:** Univariate analysis of risk predictors of cardiovascular complications

**Variables**	**Cardiovascular complication**		**OR**	**95%CI**	**p Value**
	**Present(%)**	**Absent (%)**			
Drug treatment*	35.4	65.6	4.57	1.84-11.35	0.001
Maternal smoking	29.4	70.6	1.86	0.59-5.86	0.327
SAH	13.0	87.0	0.56	0.15-2.05	0.295
Preeclampsia	08.3	91.7	0.34	0.43-2.80	0.450
LHO	60.9	39.1	3.04	1.35-6.86	0.018
Previous cardiac complications	38.6	61.4	3.65	1.59-8.41	0.002
EF > 60%	18.5	81.5	2.38	1.34-5.42	0.038
NYHA class III	25.0	75.0	3.89	1.23-7.69	0.011
HRPC initiated on the 3rd trimester	15.4	84.6	1.34	0.50-3.57	0.379
Valve prosthesis	21.1	79.0	1.10	0.33-3.65	0.542

* Need to initiate/change cardiac medication. OR: odds ratio; 95%CI: 95% confidence interval; SAH: systemic arterial hypertension; LHO: left-heart obstruction; EF: ejection fraction; HRPC: high-risk prenatal care; CARPREG: Cardiac Disease in Pregnancy.

On multivariate analysis, the following factors were independent risk predictors of cardiovascular complications that can influence maternal-fetal outcomes: need to initiate or change cardiac medication during pregnancy [p = 0.009; 95% confidence interval (95%CI): 0.058-0.408]; previous cardiac complications (p = 0.013; 95%CI: 0.401-0.342); and functional class III on the first prenatal visit (p=0.041; 95%CI: 0.032-0.134).

The perinatal outcomes assessed in 129 pregnancies were as follows: 13 (10.07%) small for gestational age newborns and 34 (26.36%) premature babies (4 aged less than 30 weeks, 14 between 32 and 34 weeks, and 16 between 35 and 37 weeks). No association was found between those results and risk factors for maternal cardiovascular complications.

## Discussion

The present study describes the profile of a population of pregnant women with heart disease, mainly rheumatic lesions, which are usual in the Brazilian population. A 22.72% prevalence of cardiovascular complications in pregnancy was found, a rate close to those found in this same institution in 1997^7^ and at the Instituto do Coração (Incor) of the Medical School of the São Paulo University,^8^ 23.9% and 23.5%, respectively. The most recent international studies have revealed a lower number of complications: a Canadian study has reported 13% of complications;^4^ the ZAHARA I study, conducted in Holland in 2010,^5^ reported a 7.6% incidence; and, even more recently, in 2013, an European collaborative study reported 10% of cardiovascular complications.^9^ That difference in the complication rates as compared to international data can be partially explained by the difference in the characteristics of the populations studied. In addition, pregnant women with heart disease in developed countries are more likely to have earlier and easier access to prenatal follow-up, which did not happen in 75% of this study population.

This study shows that the population of pregnant women with heart disease cared for at our institution did not change much in the past 17 years regarding etiology. Over half of this population (62%) had rheumatic heart disease, a percentage similar to that obtained by Bacha et al.^7^ (56.8%) from 1990 to 1995. That percentage was also close to the one reported by Ávila et al.^8^ when following 1,000 patients up at Incor during a similar period.

Of the assessments for risk prediction of cardiovascular complications during pregnancy, only Siu et al.^4^ and Tanous et al.^10^ have included both congenital and acquired cardiac diseases, but with a greater prevalence of congenital heart diseases (74% in both studies). In other studies, all populations analyzed consisted of pregnant women with only congenital heart diseases.^6,10-12^ Of the complications presented by our patients, the most frequent was cardiac decompensation (11.36%), a diagnosis that can be inaccurate, because, during pregnancy, the distinction between the physiological changes inherent in pregnancy and the signs of heart disease is difficult. The criteria used to consider cardiac decompensation, as discussed in the ZAHARA study,^5^ need to be better defined and can explain the elevated percentage of complications in our study population as compared to that of other publications.^2,6^

### Comparison with the CARPREG risk score

Although the total frequency of complications was greater in our patients, when assessing the incidence of cardiovascular complications by using the CARPREG risk score, risk overestimation was observed in the pregnant women classified as CARPREG 1 and > 1. Our patients classified as CARPREG 1 had a 16.42% rate of complications as compared to the 27% proposed by the study by Siu et al.^4^ Those classified as CARPREG > 1 had a 42.11% rate of complications as compared to the 75% expected according to the same index. This can be due to the apparent lower severity of the heart diseases in our population of pregnant women.

Similarly, the LHO in our population less often progressed to cardiac decompensation or other complications. While the mean mitral valve area in our group of patients was 1.62 cm^2^ and that of the aortic valve was 1.4 cm^2^, those of the pregnant women followed up in the CARPREG study^4^ were smaller, 1.3 and 0.9 cm^2^, respectively.

The congenital diseases of the patients in this study, in addition to being less frequent, were less complex, reflecting the reduced number of patients with ventricular dysfunction and complex congenital heart diseases who reach the fertile age in the Brazilian population.

Although the patients classified as CARPREG 1 and > 1 had a lower percentage of complications than expected, the CARPREG 0 group had twice more complications than that expected according to the CARPREG risk score^2^ (11.36%). This risk underestimation can reflect a late diagnosis of heart diseases in young women, pregnancy being the moment of the first diagnosis.

Other studies conducted with other populations have also reported an overestimation of the risk for complications by the CARPREG risk score. The authors of the ZAHARA I study^5^ have attributed that overestimation by the CARPREG risk score to the possibility that patients with acquired heart diseases had more severe lesions in the CARPREG study than in other studies and also to the criteria used to define cardiac decompensation. Tanous et al.^10^ and Curtis et al.^13^ have also observed overestimation by the CARPREG risk score when using it in their patients, and have suggested that population differences would account for that.

The ZAHARA II study^6^ has considered that CARPREG has a high prediction power for cardiac events in patients at moderate and high risk, but that it could underestimate the risk in patients classified as low risk.

Regarding the presence of advanced functional class at the beginning of pregnancy, all studies, regardless of the population studied, indicate that variable as a risk predictor, similarly to that observed in our population. Since the studies by Bacha et al.,^7^ that association of maternal complications has been reported in the presence of a baseline NYHA classification III or IV at the beginning of the prenatal care or when the pregnant woman has pulmonary hipertension.^2,4,8,12^

Maternal smoking did not prove to be an independent risk factor for maternal complications, which is in accordance with the observation of other authors.^4,6^ Khairy et al.,^11^ however, have found an association of maternal smoking with maternal complications, indicating that a more careful interpretation is required regarding that habit.

The variables identified on multivariate analysis as predictors of complication in that population are very evident on clinical practice. The need to initiate or change maternal medications during pregnancy was associated with maternal complications (odds ratio of 4.57), and can be interpreted as an equivalent to the need for intervention due to NYHA functional class worsening during the pregnancy-puerperal cycle.^8,12^

Other risk factors proposed in the CARPREG study, such as LHO, ventricular dysfunction and previous cardiac complications, were associated with maternal complications on univariate analysis, but were not considered significant in the logistic regression model^13^. The reduced number of patients with that condition in our study could explain that difference. This analysis suggests that, in populations in which rheumatic acquired heart diseases prevail, the LHO conditions, which actually predict cardiovascular complications in pregnancy, are those with mitral stenosis, mainly in the presence of severe valve area reduction.

Regarding perinatal outcomes, approximately one quarter of the newborns were premature and/or small for the gestational age, which is usually the most direct complication of severe maternal complications, which lead to premature interruptions of pregnancy and a reduction in placental nutrition. However, of that population, only 4 newborns were extremely premature, and, probably because of that small number, we could not find the expected association with maternal outcomes.^14^

### Study limitations

The obstetric factors were not controlled, which can have influenced the results of the present study.

Siu et al.^4^ have reported that, even in the presence of LHO, cyanosis and advanced NYHA functional class, women with heart disease and no other obstetric risk factor had a minimally increased risk of neonatal complications. In addition, no patient had cyanosis, and the number of women with advanced functional class was relatively small.

### Clinical implications

The present study emphasizes the need for the early assessment of heart disease in pregnancy, that is, in young women. Our patients' mean age was 27 years, in accordance with the mean age described in almost all international studies. However, most of our patients arrive at the HRPC outpatient clinic from the second trimester on, and 40%, after the 20th gestational week. Those data show that our patients are referred to the reference center later. Regarding the quality of the patients' follow-up care and of the family planning offered, it is worth noting that most women were between their second and fifth pregnancy, and almost 12% of them were at least on their fifth pregnancy. Comparison with studies from developed countries, such as that by Siu et al.,^4^ in which only 1% of the women were on their fifth pregnancy and most (58%) of them were on their first pregnancy, evidences the great difference between developed and developing countries regarding the prevention of cardiovascular complications based on effective prenatal counseling and family planning. Our patients most likely do not have a regular follow-up with a cardiologist, and, thus, receive poor information on the risks of pregnancy regarding their cardiac problems. Ideally, pregnancy should be fully planned to occur on an occasion of disease stability, and the obstetric follow-up should be initiated on the first trimester.^13,15^

Most severe rheumatic diseases should be treated, usually with invasive procedures, before pregnancy. This would reduce the need to use those procedures during pregnancy itself, diminishing maternal-fetal morbidity and mortality.^16,17^ Other patients with more severe forms and no possibility of effective treatment should be oriented to avoid pregnancy and should receive effective contraceptive counseling.^15,18^

The intermediate- and long-term prospective follow-up of a significant number of patients with severe heart diseases can provide a more adequate analysis of the near miss situations. In addition, it will enable the assessment of the disease impact on the quality of life, sexual and reproductive health, and long-term consequences of the overload pregnancy imposes on patients with impaired cardiac function. It will also contribute to the appearance of health policies aimed at that group of patients.

## Conclusion

In this study on pregnant women with heart disease, mostly rheumatic heart disease, the following independent risk factors for cardiovascular complications during pregnancy stood out: beginning or changing cardiac medication during pregnancy; cardiac complications prior to the gestational period; and NYHA functional class III at the beginning of prenatal follow-up. In addition, the use of CARPREG risk score in that population tended to underestimate the risk of patients classified as low risk and to overestimate the risk of those classified as moderate or high risk.
